# *Lactobacillus gasseri* Causing Bilateral Empyema

**DOI:** 10.1155/2017/4895619

**Published:** 2017-09-25

**Authors:** Angela Esquibel, Ala S. Dababneh, Bharath Raj Palraj

**Affiliations:** ^1^Division of Family Medicine, Mayo Clinic Health System, 800 West Avenue South, La Crosse, WI 54601, USA; ^2^Division of Infectious Diseases, Mayo Clinic, 200 First Street SW, Rochester, MN 55905, USA

## Abstract

Lactobacilli are common commensal bacteria found in the gastrointestinal and genitourinary tract. Although they are usually thought to be nonpathogenic, there have been several cases that demonstrate severe infections caused by these microorganisms. This is a case of a 49-year-old male with previously undiagnosed type two diabetes mellitus who presented with a 3-month history of cough and was found to have right sided* Lactobacillus gasseri* empyema for which he underwent video-assisted thoracoscopic surgery (VATS) with chest tube placement. He subsequently developed a left sided pleural empyema for which the aspiration also grew out* L. gasseri*. The patient made a complete recovery and was seen for four months in follow-up after his initial presentation.

## 1. Introduction

*Lactobacillus gasseri* is a member of the Lactobacillaceae family, a commensal organism of the gastrointestinal and genitourinary tract. It is rarely encountered as a cause of clinically significant infections. Previously reported cases include pneumonia, urinary tract infection, and polymicrobial empyema. In this case, we are presenting the first case of a monomicrobial* Lactobacillus gasseri* bilateral empyema that was successfully treated with source control and pathogen directed antibiotic therapy.

## 2. Case Presentation

**Figure 1:**
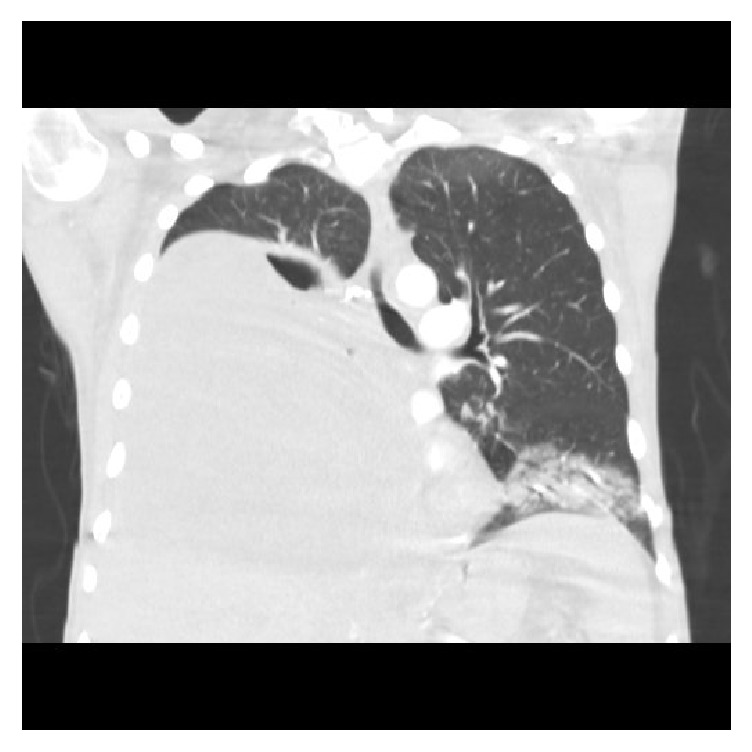
CT on admission showing a large right sided pleural effusion containing pockets of gas along with focal airspace disease within the lingula and left lower lobe.

**Figure 2:**
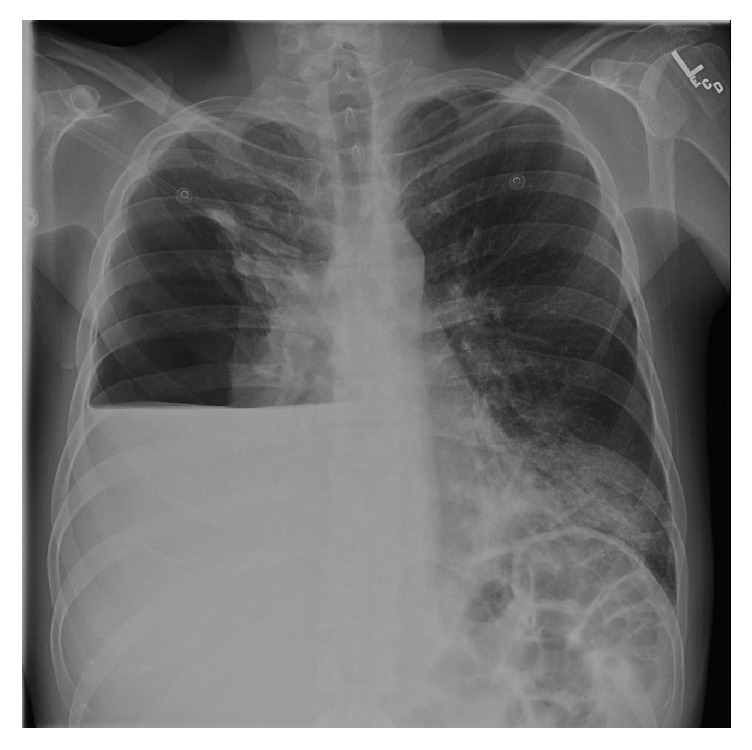
Chest X-ray after thoracentesis showing large right pneumothorax with collapse of the lung into the hilar region. Large right hemithorax air-fluid level. Increased airspace consolidation within the left lower lobe.

This is a previously healthy 49-year-old male who had a 3-month history of cough and worsening fatigue. The cough was productive for yellow brown sputum and was associated with a 30-pound weight loss. The patient also noted excessive lower extremity edema. He denied any smoking history and denied any respiratory conditions. Patient denied any industrial work or exposure to asbestos or other carcinogenic materials. Patient denied any personal history of cancer. He denied any prior history of diabetes or high blood sugars. He had no known medical problems and did not see a physician regularly. Upon presentation, he was acutely hypoxic saturating 93% on 4 L of O2 nasal cannula and tachycardic. On physical examination, his entire right lung sounds were diminished, with mild crackles noted bilaterally. Lower extremities showed 2 to 3+ pitting edema bilaterally without obvious erythema or weeping. Laboratory findings included an elevated white count of 23.8 × 10(9)/L, sodium of 128 mM/L, potassium of 4.4 mmol/L, chloride of 79 mmol/L, CO2 of 20 mmol/L, BUN of 21 mg/dL, creatinine of 0.57 mg/dL, and glucose of 453 mg/dL. Liver function tests were within normal limits. Lactic acid was 1.7 mg/dl. Hemoglobin A1c was 14.7%. Hemoglobin was 11.4 g/dL. Platelets were 476 × 10(9)/L. HIV test was negative. CT scan of the chest was done and showed a large right sided pleural effusion containing pockets of gas along with focal airspace disease within the lingula and left lower lobe (Figure 1). Chest X-ray performed after thoracentesis showed a large right pneumothorax (Figure 2).

**Figure 3:**
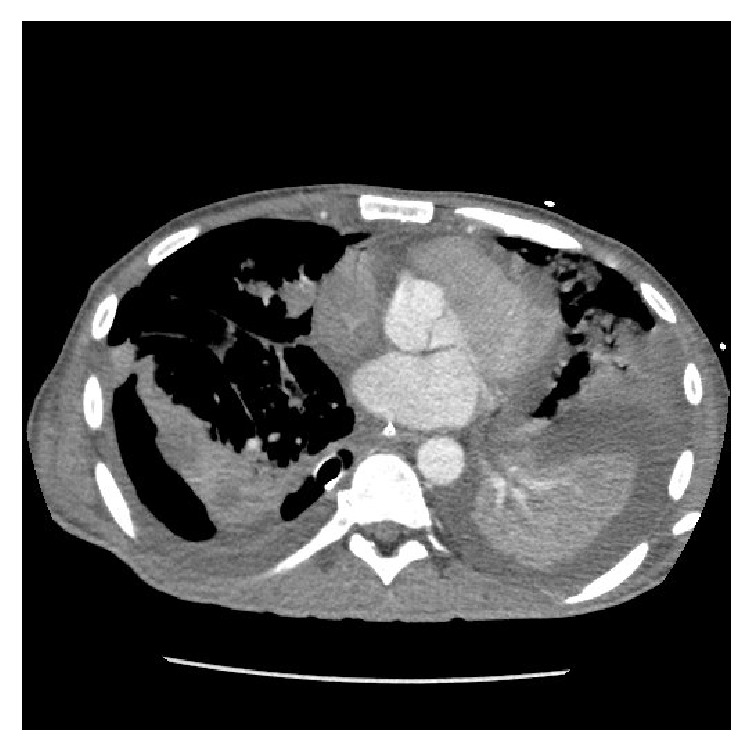
Left sided empyema and left lower lobe collapse prior to drainage.

He was placed on medical therapy for likely mild to moderate DKA along with broad spectrum antibiotic coverage with piperacillin-tazobactam (Zosyn), levofloxacin, and vancomycin. He underwent a right sided VATS procedure for drainage of the empyema and two chest tubes were placed. The right chest was entered through a more posterior incision where a large abscess cavity draining approximately 1300 mL of gray foul-smelling material was encountered. Pleural biopsy and cultures were taken. Initial sputum culture from admission demonstrated* Candida* species and many Gram-positive rods resembling Lactobacilli. Cultures from the pleural fluid obtained during the VATS procedure grew* Lactobacillus gasseri*. Antibiotics were deescalated to piperacillin-tazobactam alone. Postoperatively his course was complicated by acute respiratory distress syndrome (ARDS) and repeated CT chest imaging showed left sided empyema and left lower lobe collapse (Figure 3) and a left chest tube was placed for drainage on hospital day 8. Left lung pleural fluid cultures also grew* L. gasseri*. The laboratory methods for the sputum culture and pleural fluid aerobic culture (from both right and left lung pleural fluid) were a send-out test to a larger teaching hospital that our community hospital is affiliated with. Both cultures were identified as GP bacilli, alpha colony*, Lactobacillus gasseri* susceptible to penicillin [1]. Pleural biopsy revealed reactive mesothelial cells and acute and chronic inflammation.

**Figure 4:**
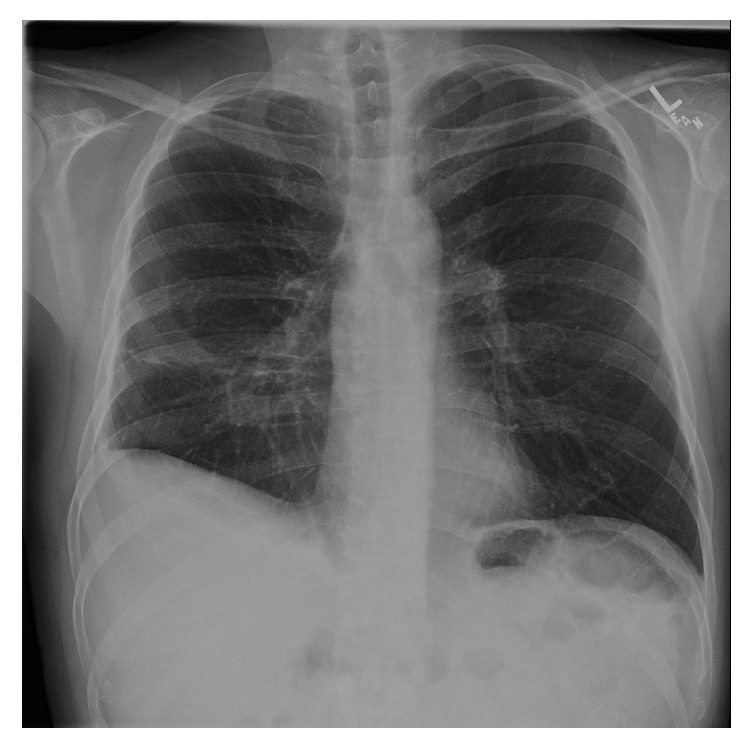
4 months after hospital discharge.

On hospital day 10 he was weaned off of pressor support and underwent a tracheostomy and PEG placement. Patient continued to improve and was transitioned to tracheostomy collar by hospital day 13. Antibiotics were changed to Ampicillin/Sulbactam for the remainder of his hospital stay. Patient was discharged on hospital day 25 on Amoxicillin-clavulanate 875 mg/125 mg. On discharge he had one remaining chest tube on the right side, and there was a persistent bronchopleural fistula. Seven weeks after his initial presentation his chest tube was removed. His oral antibiotics were discontinued shortly thereafter. Chest X-ray was obtained four months after his hospital discharge (Figure 4).

## 3. Discussion

Lactobacillaceae are non-spore-forming, strictly anaerobic or facultative, Gram-positive bacilli that normally colonized in oral cavity, gastrointestinal tract, and female genital tract [1, 2].* Lactobacillus gasseri* is a member of the Lactobacillaceae family which was first identified in 1980 and prior to that was named* L. acidophilus *[1, 2]. It thrives in anaerobic conditions and represents the major* Lactobacillus* species in the gut and plays a major role in maintaining a healthy bowel flora [1]. It is a non-spore-forming Gram-positive rod that is typically resistant to vancomycin and forms lactic acid from glucose [2]. In the food industry, it plays a role in fermentation of food products and is rarely pathogenic in humans [3]. Infectious cases reported in the literature cover a diverse span of clinical conditions including life-threatening conditions such as endocarditis [4], meningitis [5], splenic abscess [6], empyema and pneumonia [7], emphysematous pyelonephritis [8], peritonitis [9], and Fournier gangrene [2]. Many of the reported cases were associated with immunosuppressive conditions such as cancer, chemotherapy induced neutropenia, long term steroids, peritoneal dialysis, diabetes, transplantation, long term antibiotics, or postoperative complication. The most frequently associated infections with* Lactobacillus* are bacteremia and endocarditis, and* L. casei* and* L. rhamnosus *were most commonly encountered [10]. Infections by* Lactobacillus gasseri* are rarely reported in the literature. A case of Fournier gangrene [2], urinary tract infection [11], and polymicrobial empyema [7] were previously reported. In this case, we present the first monomicrobial* Lactobacillus gasseri* bilateral empyema that was successfully treated by video-assisted thoracoscopic surgery along with chest tube placement and drainage. Identification of the* Lactobacillus* species was made using matrix-assisted laser desorption ionization time-of-flight mass spectrometry which was done at a reference laboratory [12]. Pneumonia and subsequent empyema were the two most fatal diseases encountered in ancient time [13]. Hippocrates is credited with both naming and inventing the surgical technique for proper management [13]. Empyema remains a serious medical condition with associated morbidity and mortality. Common pathogens that are encountered include* Streptococcus*,* Staphylococcus*,* Enterobacteriaceae*, and anaerobes [14]. Despite the introduction of antibiotic therapy and the application of the scientific method to the modern practice of medicine when it comes to managing empyema, the core principle is still adequate drainage.

The causative organism for his empyema is* Lactobacillus gasseri* as this was the only organism that was isolated from his pleural effusion cultures, along with his sputum culture; although* Candida albicans* was also isolated from his sputum sample, it likely reflects oral colonization. Prior to presentation, the patient had no prior antibiotic exposure and did not frequently seek medical care. The likely mechanism for this infection is aspiration in the setting of undiagnosed diabetes type 2 with Hgb A1c of 14.2%. Management requires adequate drainage along with adequate antibiotic coverage guided by culture results. The duration of therapy is variable and depends on host factors and rate of clinical improvement.

## 4. Conclusion

In conclusion,* Lactobacillus gasseri* is a rare cause of empyema. Management of empyema requires adequate source control along with culture directed antibiotic therapy. Despite the tendency to consider Lactobacilli as an uncommon pathogen, the identification of* Lactobacillus gasseri* in extraintestinal or extragenitourinary sites of infection should not be overlooked.
